# High Intensity Microsecond-Electric Pulses Modulate Cell Death in MDA-MB-231 Cells Treated with Resveratrol

**DOI:** 10.1007/s12013-026-02075-3

**Published:** 2026-04-17

**Authors:** Pragatheiswar Giri, Ignacio Camarillo, Elisabetta Sieni, Raji Sundararajan

**Affiliations:** 1 School of Engineering Technology, Purdue University, West Lafayette, IN 47907 USA; 2 Dept. of Biological Sciences, Purdue University, West Lafayette, IN 47907 USA; 3 Dept. of Applied and Theoretical Science, University of Insubria, Via Dunant 3, Varese 21100 Italy

**Keywords:** Resveratrol, MDA-MB-231, Electroporation, ROS

## Abstract

In this study, we investigated the potential of high-intensity microsecond electrical pulses (EPs) to augment intracellular delivery and enhanced uptake of Resveratrol (Resv), a natural polyphenol, as a novel therapeutic agent in the treatment of Triple Negative Breast Cancer (TNBC). For this purpose, MDA-MB-231, human TNBC cells were used. Resv exhibits potent anticancer properties, including the inhibition of tumor initiation, promotion, and progression, by modulating key molecular pathways involved in cell growth and apoptosis. Its multifaceted mechanisms of action make it a promising anticancer agent, especially for TNBC, characterized for its aggressive nature and high mortality rates, posing significant challenges in oncology. Conventional treatment modalities often are refractive, due to the absence of the most common hormone receptors, leading to increased recurrence and metastasis. Our approach leverages the bioelectrical modulation capacity of EPs to transiently permeabilize the TNBC cell membranes, thereby enhancing the uptake of Resv, a natural compound with limited bioavailability. Utilizing the MDA-MB-231 TNBC cell line, the impact of varying EP parameters (8 pulses at electric field of 2500 V/cm to 5000 V/cm, 10µs, 1 Hz) on cell viability and intracellular reactive oxygen species (ROS) were explored. Results indicated cell survival, as low as 11%, at 48 h with 5000 V/cm pulses, while it was 15% for 2500 V/cm (control at 100%). The ROS levels were enhanced to 350% (control at 100%). The consequent apoptotic activity due to ROS underscore the potential of this novel bioelectrical modulation technique in improving the outcomes of TNBC treatments. This research contributes to the emerging field of electrical pulse modulation in enhanced drug delivery and offers a promising avenue for addressing the limitations of current TNBC therapeutic strategies.

## Introduction

Triple-negative breast cancer (TNBC) constitutes approximately 15–20% of newly diagnosed breast malignancies, yet is responsible for a disproportionate higher share of disease-specific mortality owing to its intrinsically aggressive phenotype and paucity of actionable molecular targets [1–3]. Genomically, TNBC exhibits a basal-like transcriptome, high mutational burden driven largely by TP53 and BRCA1/2 lesions, pervasive chromosomal instability, and frequent activation of MYC, PI3K-Akt, and Wnt/β-catenin pathways [4]. Phenotypically, these tumors display elevated epithelial–mesenchymal plasticity, cancer-stem-cell (CSC) enrichment, and a predilection for visceral and cerebral metastasis within three years of primary therapy [5]. Current standard of care using anthracycline-based chemotherapy, with or without platinum salts yields modest pathological complete-response rates, while recently approved immune checkpoint blockade benefits only a PD-L1-positive subset and is frequently thwarted by compensatory immunosuppressive pathways [6]. Consequently, median overall survival in metastatic TNBC remains < 18 months, underscoring the imperative for mechanism diverse adjuvant modalities [7]. Considering the above limitations and side effects brought about by the toxic chemotherapeutics, in this research, we investigated the natural compound, Resveratrol.

Resveratrol (3,5,4′-trihydroxy-trans-stilbene), a naturally occurring stilbenoid polyphenol, has garnered attention for its pleiotropic anticancer properties, such as cell-cycle arrest, anti-angiogenic action, inflammation modulation, chemo/radio sensitization. In pre-clinical breast-cancer models, resveratrol reactivates p53, attenuates PI3K/Akt/mTOR and EGFR/ERK axes, suppresses NF-κB-driven pro-inflammatory transcription, destabilizes HIF-1α, induces S-phase arrest, and promotes both intrinsic (Bax/Bak-mediated) and extrinsic (Fas/TNF-α) apoptotic cascades [8]. Moreover, resveratrol perturbs cancer-stem-cell maintenance by down-regulating ALDH1 and Notch1 and interferes with metastatic competence via inhibition of MMP-2/9 and EMT markers. Despite this multi-targeted repertoire, clinical translation is hamstrung by physicochemical and pharmacokinetic barriers: aqueous solubility, extensive phase-II glucuronidation/sulfation in the gut–liver axis, and active extrusion by P-glycoprotein, collectively yielding sub-micromolar systemic exposures that are an order of magnitude below the in-vitro IC₅₀ required for TNBC cytotoxicity [9]. Because resveratrol has limited bioavailability and membrane transport constraints, we used electroporation that typically enhances drug uptake [10] by transiently permeabilizing the cell membrane.

Electroporation provides a biophysical solution to this bottleneck by delivering short, high-voltage pulses that elevate the transmembrane potential (Vm), beyond the dielectric breakdown threshold (~ 0.25 V for mammalian cells), nucleating transient hydrophilic nanopores (2–10 nm) in the lipid bilayer and thereby lowering the Gibbs-free-energy barrier for molecular transport [11]. Typically, short duration pules are used in EP, which temporarily disrupt the cell membrane by creating transient pores [3]. This method is particularly valuable in the field of oncology, especially for the treatment of resistant cancer types, such as TNBC, which lacks specific receptors that are typically targeted in other breast cancer treatments. Microsecond pulses have been chosen for their ability to achieve effective electroporation with minimal thermal and mechanical damage to the surrounding tissues. The rapid application and cessation of these pulses ensure that the electric fields affect the target cells’ membranes without causing significant collateral damage or prolonged stress to the cells and tissues involved [3]. Applying an external electric field displaces the charges inside and outside the cells. This leads to the accumulated transmembrane potential difference (Vm) on the cell surface. There are hydrophobic cell membrane changes, when the transmembrane potential difference exceeds the threshold value (Vm > Vth), creating microscopic, hydrophilic, and hydrophobic pores in the cell membranes. The rate of pore density is given as [12, 13]:

1 $$\:\frac{dN}{dt}=\:\alpha\:\:{e}^{({\frac{{V}_{m}}{{V}_{eq}})}^{2}}\:\left(1-\:\frac{{N}_{0}}{{N}_{eq}\left({V}_{m}\right)}\:\right)$$

where, $\:\alpha\:$ is a constant that depends on the properties of the cell membrane, V_ep_ is the voltage at which pores are created, V_m_ is the transmembrane potential, and N (t, θ) is pore density. N_eq_ is the equilibrium pore density for a given voltage V_m_, where N_0_ is the port density for V_m_ = 0 mV.

Microsecond EP has been clinically validated in electrochemotherapy (ECT) regimens that potentiate the local cytotoxicity of bleomycin and cisplatin without exacerbating systemic toxicity, achieving objective response rates exceeding 80% in cutaneous metastases [14]. Beyond merely facilitating passive diffusion, EP-induced bioelectrical perturbations can modulate calcium influx, mitochondrial membrane potential, and reactive-oxygen-species (ROS) dynamics, rendering cancer cells more susceptible to redox-active agents. Towards this, in this study, MDA-MB-231, human TNBC line was investigated as a prototypical claudin-low/basal-B TNBC model characterized by EMT features, CSC enrichment, and multi-drug resistance, using electroporation and Resveratrol, for their viability and intracellular ROS levels, that lead to cell death via apoptosis.

## Materials and Methods

### MDA-MB-231 Cell Line

MDA-MB-231 (ATCC, USA), a human Triple Negative Breast Cancer cell line, originally derived from a 51-year-old Caucasian female was used. These cells were cultured in Dulbecco’s Modified Eagle Medium (DMEM) supplemented with 10% Fetal Bovine Serum (FBS) and 1% Penicillin-Streptomycin (PS), incubated at 37 C with 5% CO2 and high humidity levels. This environment ensures the necessary support for their sustained growth and proliferation.

### Drug Preparation

4.565 mg of Resveratrol (3,5,4’-trihydroxy-trans-stilbene), Resv, sourced from Sigma-Aldrich (USA) was dissolved in DMSO to prepare a 10mM stock solution. Working concentrations of 10–150µM were tested to generate the dose-response curve and estimate the IC50. A concentration of 100 µM was selected for EP+Resv experiments, because it was near the measured IC50 and provided a suitable dynamic range for assessing treatment-related changes in viability. The vehicle control consisted of cells exposed to the same final concentration of DMSO used for resveratrol dilution, without resveratrol. The untreated control consisted of cells without DMSO or resveratrol.

### Electric Pulse Optimization

A BTX ECM 830 unipolar, square wave electroporator (Genetronics Inc., San Diego, CA, USA) was utilized to deliver eight, square-wave unipolar pulses at a frequency of 1 Hz with electric field of 2500 V/cm to 5000 V/cm and a pulse duration of 10µs. Typically, eight, 1000 V/cm, 100µs pulses are used for ECT [15–17]. Also, the electric field x time duration is a constant [18]. Hence, to study using 10µs time duration pulses, the electric field values were increased to 2500 to 5000 V/cm. 10µs duration was selected to find the impact of shorter duration pulses on these cells.

For electroporation, 4 mm gap BTX electroporation cuvettes were used with a cell suspension at a concentration of 1 × 10^6^cells/mL. All experiments were conducted in technical triplicates at a Resv dosage of 100µM.

### Cell Viability Assessment

Samples containing 1 × 10^6^cells/mL, encompassing controls and various treatments (Resv, EP1, EP2, EP3 and EP+Resv), were distributed into 96-well plates, with each well holding 55 µl of media. The RealTime-Glo™ MT Cell Viability Substrate and NanoLuc^®^ Enzyme (Promega) were prepared according to the manufacturer’s protocol. The reagent mixture was added directly to the wells at a 1:1 (v/v) ratio with the culture medium without washing or media removal. Plates were incubated at 37 °C, and luminescence was measured at 24 and 48 h. Table 1 shows the sample details.

**Table 1:** Configurational Layout of the Statistical Analysis (‘X’ marks analyzed samples)

Treatments	Statistical Layout		
	Samples	24 h	48 h
Control	1 2 3	X X X	X X X
Resv only	4 5 6	X X X	X X X
EP1 (2500 V/cm)	7 8 9	X X X	X X X
EP2 (3750 V/cm)	10 11 12	X X X	X X X
EP3 (5000 V/cm)	13 14 15	X X X	X X X
EP1 + Resv	16 17 18	X X X	X X X
EP2 + Resv	19 20 21	X X X	X X X
EP3 + Resv	22 23 24	X X X	X X X

### Reactive Oxygen Species Assessment

For ROS assay, treated TNBC cells were seeded in a 96-well plate with 20 µl of the test sample and 60 µl of media. Subsequently, 20 µl of the H₂O₂ substrate (ROS-Glo™, Promega) was added. Following a total incubation period of 24 h, including 6 h post-substrate addition, 100 µl of luciferase detection reagent was introduced to each well. Luminescence in Relative Light Units (RLU) was recorded after 20 min.

### Statistical Analysis

Data are presented as mean ± SEM from three replicate samples (*n* = 3). Statistical analyses were performed using GraphPad Prism v10.1.1. For single-time-point datasets, differences among groups were analyzed by one-way ANOVA followed by Tukey’s multiple-comparison test. For the viability dataset containing two time points (24 h and 48 h), statistical analysis was performed using ordinary two-way ANOVA including treatment and time as factors, followed by Šidak’s multiple-comparison test for selected pairwise comparisons within each time point. A value of *P* < 0.05 was considered statistically significant. Significance was denoted as ns, *P* ≥ 0.05; * *P* < 0.05; ** *P* < 0.01; *** *P* < 0.001; **** *P* < 0.0001.

## Results

### Dose-response curve

**Fig. 1:**
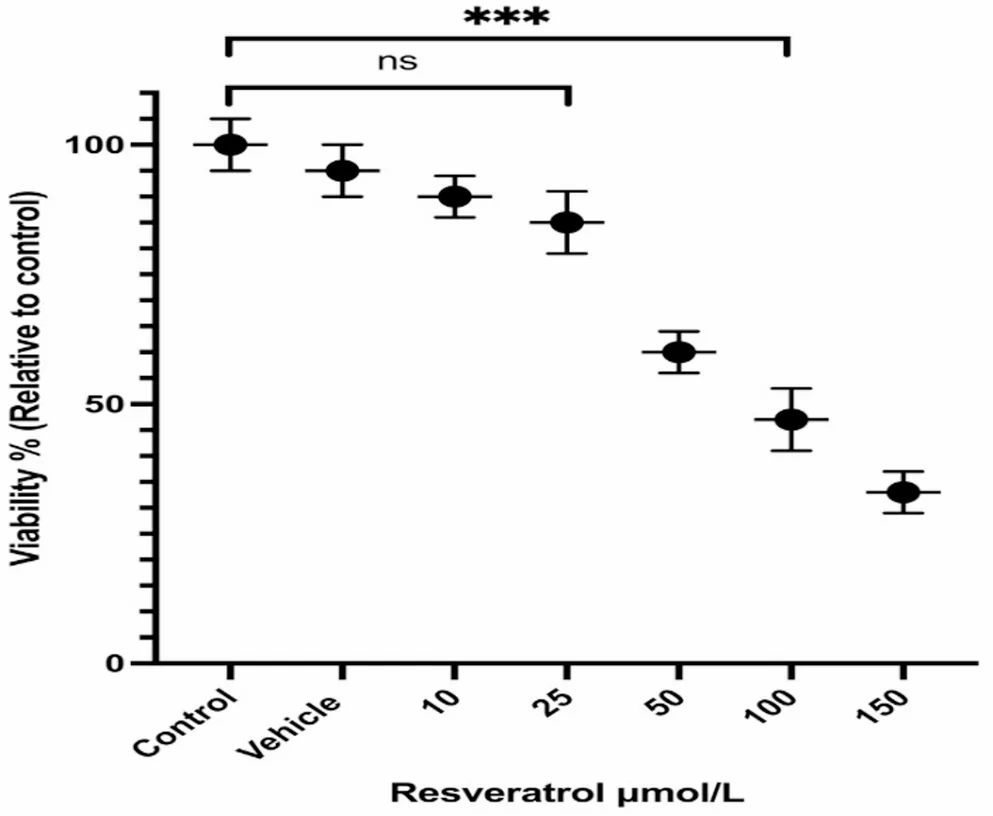
Dose-response curve of resveratrol (Resv) in MDA-MB-231 cells at 24 h. Cell viability was assessed using the Promega RealTime-Glo MT Cell Viability assay and normalized to the untreated control (= 100%). Vehicle denotes cells treated with DMSO alone without Resv. Data are presented as mean ± SEM (n = 3). Significance was denoted as ns, P ≥ 0.05; * P < 0.05; ** P < 0.01; *** P < 0.001; **** P < 0.0001

Figure 1 illustrates the dose-response curve of Resv on the viability of MDA-MB-231 cells at 24 h. Here, the viability of control was normalized to 100%. There is a clear dose-dependent reduction in cell viability. At a lower dose of 10µM, the viability was 88%, while increasing the dose to 25µM resulted in a decreased viability of 67%. At higher doses of 100µM and 150µM, the viability further reduced to 47% and 40%, respectively, and the and IC₅₀ was 63.06µM. A dose of 100µM was chosen for further experiments, as it was the first concentration after the IC₅₀value. Table 2 shows the statistical summary at 24 h.

**Table 2:** Statistical summary for resveratrol dose–response analysis in MDA-MB-231 cells at 24 h

Comparison	Adjusted *P* value	*P* value summary	Significant?
Control vs. 100µM Resv	0.0003	***	Yes

### Electric Pulse optimization Curve

**Fig. 2:**
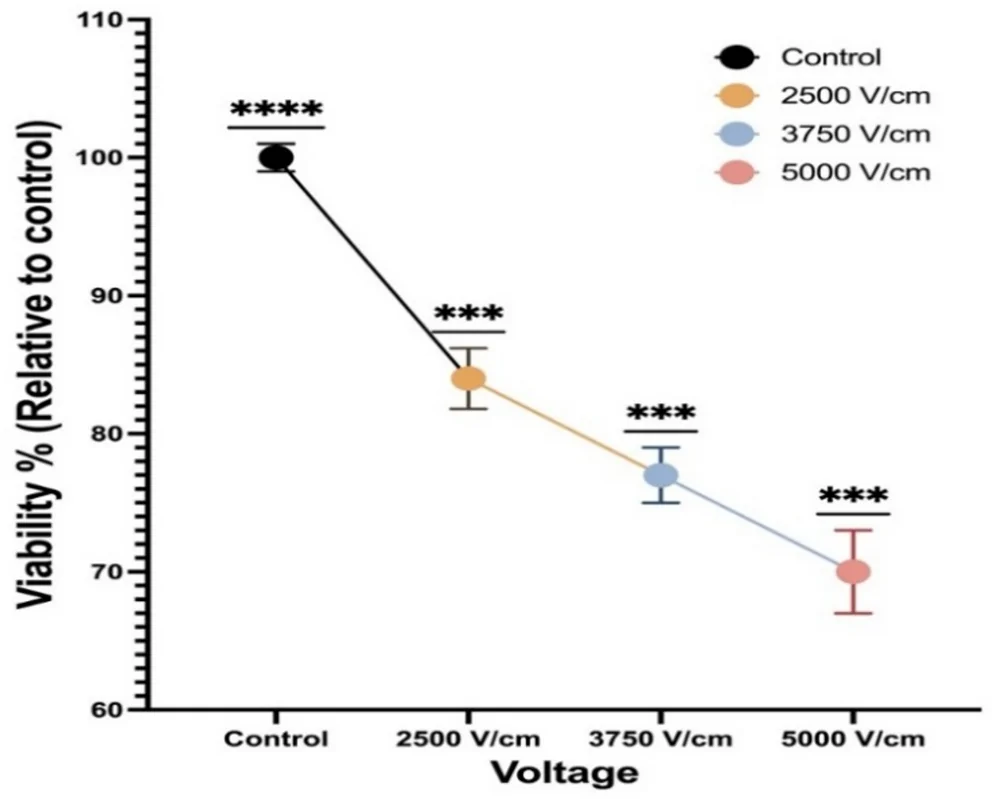
Cell viability of MDA-MB-231 cells following exposure to various electric field strengths aimed at determining the optimal parameters for Electroporation (EP) treatments

The impact of electrical pulses on the cell viability at 24 h post-treatment is depicted in Fig. 2, where control viability was normalized to 100%. There is a noticeable inverse correlation between electric field intensity and cellular survival; as the field strength increases, cell viability decreases. Specifically, the viability was 84% at 2500 V/cm, decreased to 71% at 3750 V/cm, and further to 68% at 5000 V/cm. Table 2 shows a multiple comparison summary of electroporation only treatment effects at 24 h.

**Table 3:** Multiple-comparison summary for electroporation-only treatment effects on MDA-MB-231 cell viability at 24 h. Significance was denoted as ns, *P* ≥ 0.05; * *P* < 0.05; ** *P* < 0.01; *** *P* < 0.001; **** *P* < 0.0001

Comparison	Adjusted *P* value	Significance
Control vs. EP1	0.0002	***
Control vs. EP2	0.0002	***
Control vs. EP3	0.0006	***

### Cell Viabilities with EP+Resv

**Fig. 3:**
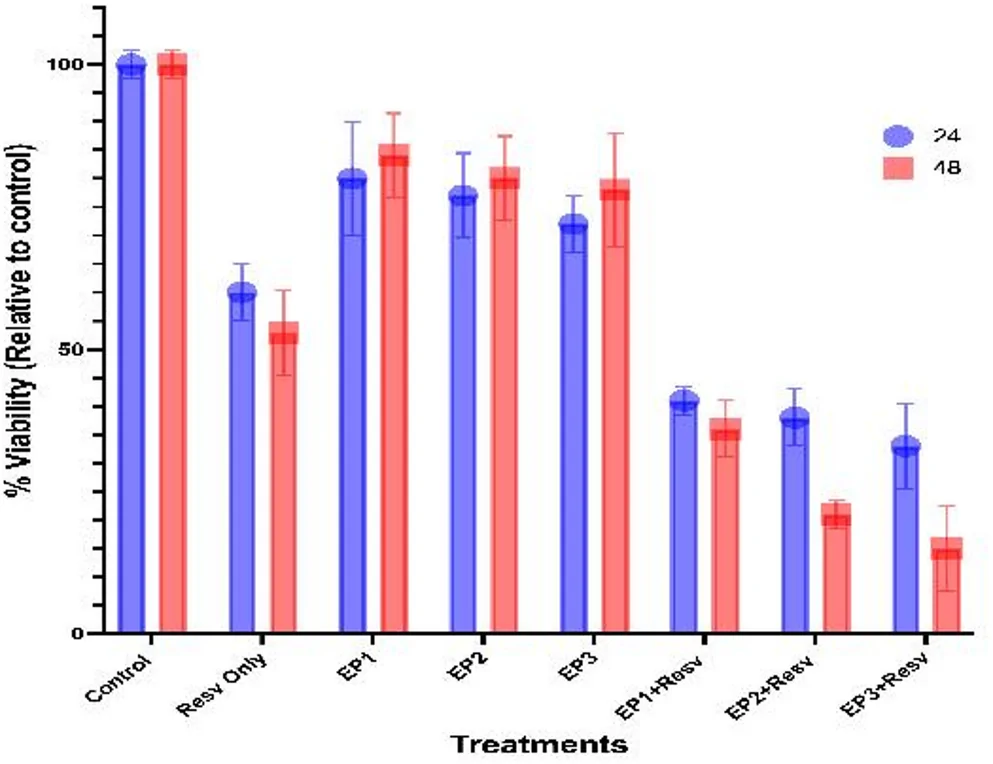
The viability of MDA-MB-231 cells under different conditions at 24h and 48h time intervals: No treatments (Control), 100µM Resveratrol (Resv only), EP1 (2500V/cm) , EP2 (3750V/cm), EP3 (5000V/cm), EP1+Resveratrol (EP1+Resv), EP2+Resveratrol (EP2+Resv), EP3+Resveratrol (EP3+Resv)

Figure 3 illustrates the effects of different EP parameters (8 pulses at 2500 V/cm, 3750 V/cm, and 5000 V/cm, each with a duration of 10µs at 1 Hz), along with 100µM Resv on cell viability. The results show that the lowest cell survival rate, 11%, was induced by 5000 V/cm pulses, followed by 15% at 3750 V/cm, and a 21% reduction at 2500 V/cm. Notably, an enhanced effect was observed when combining 100µM Resveratrol with microsecond electroporation treatments across various time points (24 h, and 48 h), leading to a significant decrease in cell viability compared to control. While cells treated solely with EP initially showed a decrease in viability at 24 h, this effect did not persist, as viability began to increase post-24 h, indicating that the effect of EP alone was less sustained over time, indicating the anticancer efficacy achieved through the combined use of Resveratrol and EP. Table 3 shows the Two-way ANOVA summary for cell viability across various treatment groups and time points.

**Table 4:** Two-way ANOVA summary for cell viability in MDA-MB-231 cells across treatment groups and time points (24 h and 48 h). Significance was denoted as ns, *P* ≥ 0.05; * *P* < 0.05; ** *P* < 0.01; *** *P* < 0.001; **** *P* < 0.0001

Source of Variation	% of total variation	*P* value	*P* value summary	Significant?
Interaction	2.708	< 0.0001	****	Yes
Row Factor	0.6483	< 0.0001	****	Yes
Column Factor	96.01	< 0.0001	****	Yes

**Fig. 4:**
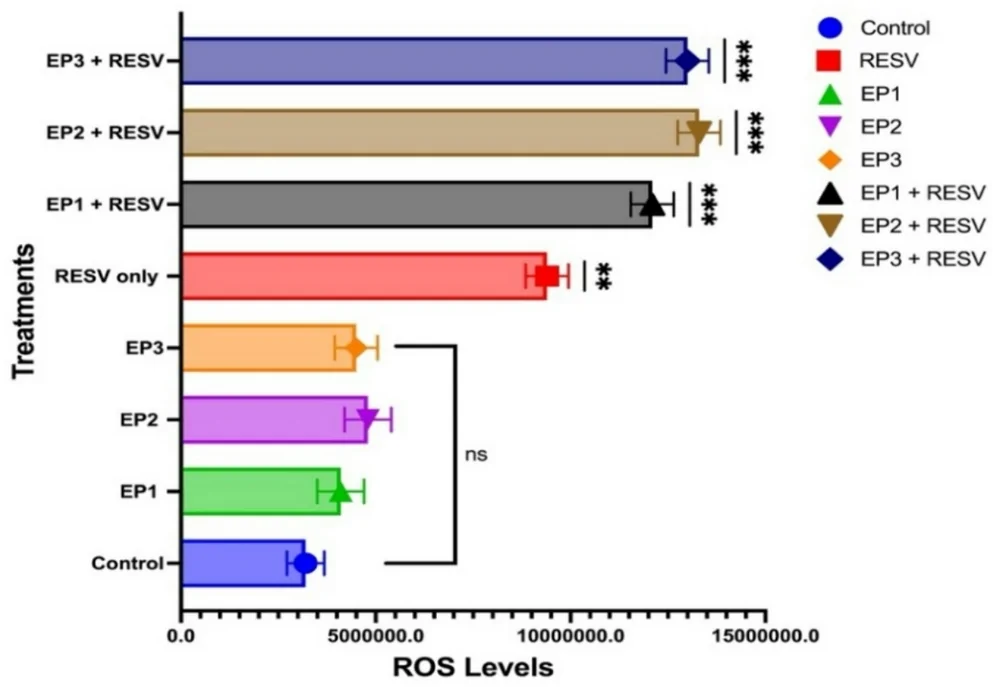
The viability of MDA-MB-231 cells under different conditions at 24 h and 48 h time intervals: No treatments (Control), 100µM Resveratrol (Resv only), EP1 (2500 V/cm), EP2 (3750 V/cm), EP3 (5000 V/cm), EP1 + Resveratrol (EP1 + Resv), EP2 + Resveratrol (EP2 + Resv), EP3 + Resveratrol (EP3 + Resv)

### Reactive Oxygen Species

Figure 4 depicts ROS levels in MDA-MB-231 cells following different treatments. The combination of EP2 and Resv at 100 µM resulted in a 3.5x increase in ROS. While Resv alone elevated ROS levels, the increase was significantly lower than that observed with EP+Resv. EP alone had minimal impact on intracellular ROS levels, highlighting the pronounced effect of the combined treatment. This substantial ROS elevation induces oxidative stress in MDA-MB-231 cells. Table 4 shows the multiple comparison summary for the ROS levels at 24 h.

**Fig. 5:**
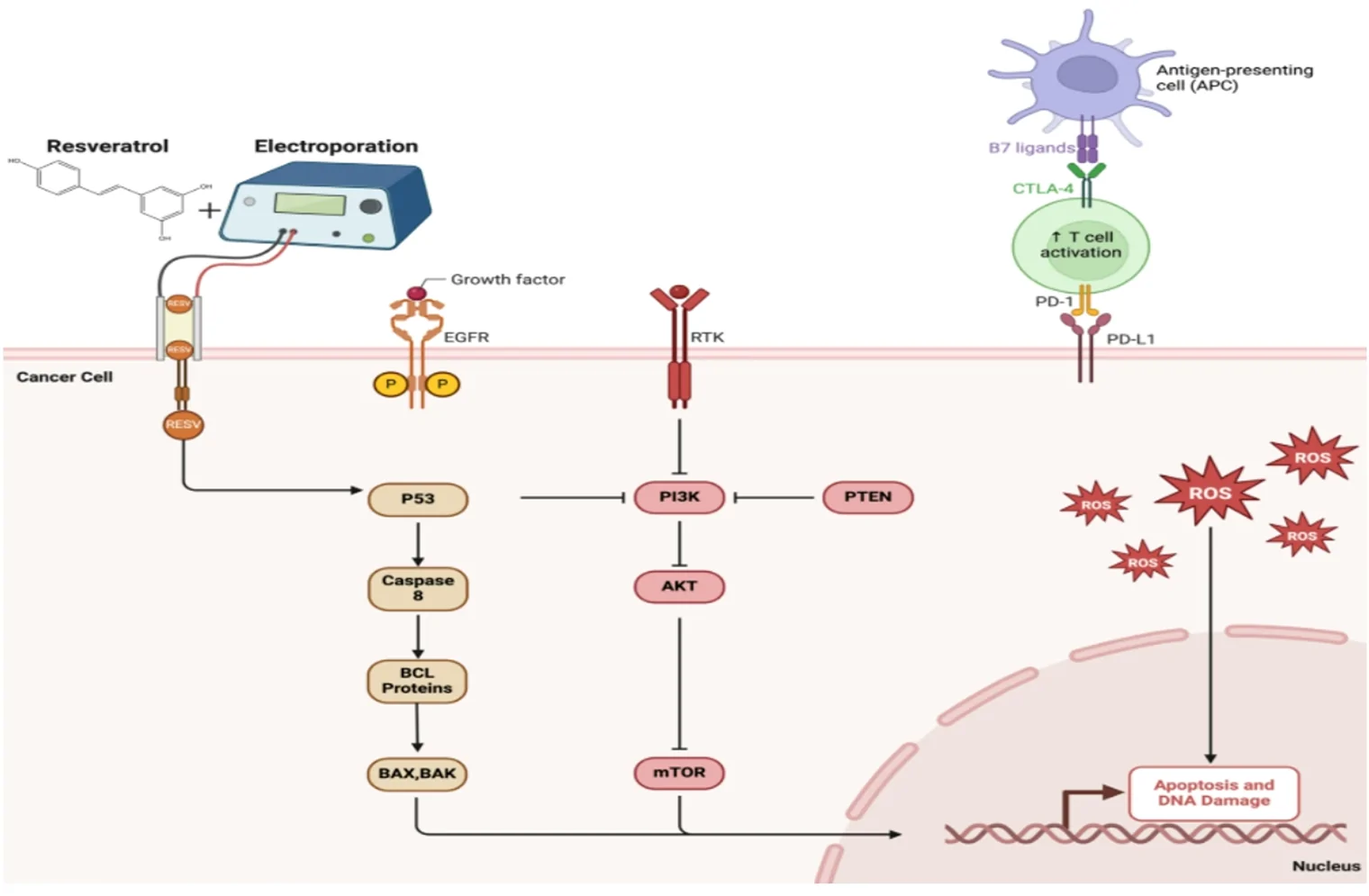
Illustration of hypothetical mechanism of due to Resv and Electric pulse therapy on MDA-MB231 cells

**Table 5:** Multiple-comparison summary for intracellular ROS levels in MDA-MB-231 cells at 24 h

*P* value	0.0007	0.0006	0.0006
P value summary	***	***	***
Significant (alpha = 0.05)?	Yes	Yes	Yes

## Discussion

In this study, the modulation of the cell viability and intracellellar ROS levels, due to Resv in MDA-MB-231 TNBC cells with high-intensity microsecond EP was investigated. Across the tested conditions, the combination of EP+Resv produced a greater reduction in cell viability than either intervention alone and was accompanied by increased intracellular H_2_O_2_-associated ROS. These findings indicate that EP enhances the in vitro effect of Resv in this TNBC model. This observation is biologically consistent with both the known pharmacological profile of Resv and the established biophysical effects of electroporation. Resv has attracted considerable interest as a pleiotropic anticancer molecule, particularly because of its effects on oxidative balance, proliferative control and stress-associated cellular responses. Electroporation, through transient membrane permeabilization, provides a means of modulating cellular exposure to therapeutic agents. In this context, the present findings support the concept that electroporation can strengthen the biological response to resveratrol, as reflected by the lower viability and higher oxidative stress observed in the combination groups relative to the monotherapy conditions.

Figure 5 presents a conceptual model, summarizing how EP+Resv may interact to enhance cellular stress in MDA-MB-231 cells. Resv has been shown to modulate mitochondrial reactive oxygen species homeostasis via Sirt3-related signaling [19], while pulsed electric fields can trigger a cellular stress response associated with translational suppression through eIF2α phosphorylation and 4E-BP1 dephosphorylation [20]. In this context, the combined treatment may promote a heightened stress state, consistent with the increased ROS levels and reduced viability observed in the present work.

The viability data further show that the response to treatment was influenced by both field strength and time point. Resv alone reduced viability in a concentration-dependent manner, supporting the selection of 100µM for subsequent combination experiments. EP alone also reduced cell viability, whereas EP+Resv produced a more pronounced decline than either treatment alone, particularly at the later time point. Taken together, these findings support the interpretation that electroporation amplifies the overall cytotoxic effect of resveratrol under the present experimental conditions.

The ROS measurements provide an additional dimension to this response. Resv increased intracellular ROS relative to untreated control cells, consistent with the pro-oxidant behaviour reported for polyphenolic compounds in cancer systems. EP alone exerted a comparatively limited effect on ROS under the conditions used here, whereas the combined treatment produced a more marked increase than either monotherapy. This pattern suggests that oxidative stress is closely associated with the enhanced biological effect observed with EP+Resv. The convergence of reduced viability and elevated ROS supports the view that redox perturbation represents a central feature of the combination response in MDA-MB-231 cells.Viewed together, these findings position EP+Resv as a biologically active combination in this TNBC model. The coupling of membrane-directed physical stimulation with a redox-active small molecule appears to generate a stronger cellular response than either modality alone. This is particularly relevant in TNBC, where treatment resistance and aggressive disease biology continue to motivate the search for alternative and combinatorial therapeutic strategies. Within this framework, the present results identify microsecond EP as an effective modulator of resveratrol response and support its further development as a combination-based anticancer approach.

## Conclusions

In this research, the impact of high-intensity (2500 to 5000 V/cm), low microsecond (10µs) pulses, combined with 100 µM resveratrol was studied using MDA-MB-231, human TNBC cells. The cell viability was reduced to as low as 11% (compared to control at 100%), for 5000 V/cm at 48 h. The intracellular ROS levels enhanced to as much as 350%, compared to control at 100%. This study demonstrates the promising potential of high-intensity, microsecond electrical pulses in enhancing Resv uptake, significantly reducing TNBC cell viability and apoptosis resistance, offering a bioelectrical modulation strategy for improving therapeutic outcomes in refractory breast cancers. However, the present study does not directly quantify intracellular resveratrol uptake or define the mechanism of cell death. Further work is required to validate these findings in additional preclinical models and to assess uptake, mechanism, and translational relevance.

Study limitations include use of a single cell line, absence of pharmacokinetic profiling. These gaps will be addressed by future work. Future directions embrace (i) optimization of pulse number/spacing to maximize Resv deposition while minimizing electro‑stress, (ii) nano‑second burst superposition to target intracellular organelles, (iii) combinatorial regimens with PARP or PD‑1 inhibitors, and (iv) development of an ultrasound‑guided needle electrode array for deep‑seated primary TNBC lesions. Collectively, our findings position microsecond EP‑enabled resveratrol delivery as a mechanistically rational, locally intensified, sparing adjunct for refractory TNBC. The platform concept could be extrapolated to other poorly bioavailable phytochemicals or antibody‑drug conjugates, heralding a broader electroceutic paradigm in precision oncology.

## Data Availability

No datasets were generated or analysed during the current study.
